# Adaptive Laboratory Evolution of Microalgae: A Review of the Regulation of Growth, Stress Resistance, Metabolic Processes, and Biodegradation of Pollutants

**DOI:** 10.3389/fmicb.2021.737248

**Published:** 2021-08-18

**Authors:** Bo Zhang, Jiangyue Wu, Fanping Meng

**Affiliations:** ^1^Key Laboratory of Marine Environment and Ecology, Ministry of Education, Ocean University of China, Qingdao, China; ^2^College of Environmental Science and Engineering, Ocean University of China, Qingdao, China; ^3^National Marine Hazard Mitigation Service, Ministry of Natural Resource of the People’s Republic of China, Beijing, China

**Keywords:** adaptive laboratory evolution, microalgae, growth, metabolic regulation, biodegradation of pollutants, stress resistance

## Abstract

Adaptive laboratory evolution (ALE) experiments are a serviceable method for the industrial utilization of the microalgae, which can improve the phenotype, performance, and stability of microalgae to obtain strains containing beneficial mutations. In this article, we reviewed the research into the microalgae ALE test and assessed the improvement of microalgae growth, tolerance, metabolism, and substrate utilization by ALE. In addition, the principles of ALE and the key factors of experimental design, as well as the issues and drawbacks of the microalgae ALE method were discussed. In general, improving the efficiency of ALE and verifying the stability of ALE resulting strains are the primary problems that need to be solved in future research, making it a promising method for the application of microalgae biotechnology.

## Introduction

Microalgae are of importance in biotechnological applications, ranging from biofuel production, production of high-value by-products, bioremediation of pollutants, by virtue of their rapid growth rate, high photosynthetic efficiency, acceptable adaptability, and eco-friendly nature (Mohsenpour et al., 2021). Industrial microalgae can often withstand certain unfavorable environmental conditions such as high concentrations of phenol (Wang et al., 2016; Li et al., 2021) and flue gas (Li T. et al., 2016; Cheng et al., 2019). This highlights the significance of enhancing the environmental tolerance of microalgae before application. To date, it has been difficult to improve the specific phenotype of microalgae by genome editing technology, as the tolerance is not commonly controlled by a single gene, and most microalgae have no sequenced genome or accurate gene annotation (Zhao and Huang, 2020). On the contrary, Adaptive laboratory evolution (ALE) is a feasible method based on stress induction, which can improve the tolerance of microalgae under specific environmental stress (Zchut et al., 2003; Sandberg et al., 2019).

ALE is an attractive technique for improving the performance of microorganisms, optimizing strain phenotypes, ascertaining biological phenomena caused by the stress conditions, and latent pathway activation (Wang et al., 2016; Xiong et al., 2017; Li et al., 2021; Okurowska et al., 2021). ALE experiments began with a controlled evolution experiment on bacteria around 1950 (Novick and Szilard, 1950; Atwood et al., 1951). Thereafter, Bennett et al. (1992) and Bennett and Lenski (1993). selected *Escherichia coli* as the model organism, and successfully obtained a strain that can adapt to temperatures between 32 and 42°C after 2000 generations. In 1991, the temperature ALE tests of *Lomentaria baileyana* and *Lomentaria orcadensis* were conducted for more than 4 months. The results showed that the difference in low-temperature tolerance between the two species was caused by the difference in photoinhibition sensitivity, and there were differences in the high-temperature stability of electron transport or energy transport between the two species (Kuebler et al., 1991). Furthermore, microorganisms can divide rapidly to achieve a high cell density, and the higher mutation rate and small genome sizes mean that their mutation rates are extremely high (Gresham and Dunham, 2014). Given the speed with which beneficial mutations can arise and become fixed, the simplest way of ALE is to extend the cell culture period in the selected environment to obtain beneficial mutations by natural selection. Many research processes have “adaptively evolved” their laboratory microorganisms merely through the inevitable growth or plate inoculation cycle in cell culture. Profiting from the development of proteomics (Wang et al., 2018), transcriptomics (Perrineau et al., 2014b; Li K. et al., 2017; Qi et al., 2017; Zhou et al., 2017; Li X. et al., 2018; Sun et al., 2018a; Cheng et al., 2019; Zhu et al., 2020; Zhang et al., 2021), genomics (Perrineau et al., 2014a; Helliwell et al., 2015; Shin et al., 2017; Rossoni and Weber, 2019; Yu et al., 2020), and metabolomics (Lohbeck et al., 2014; Li X. et al., 2017; Hu et al., 2020), the application of appropriate bioinformatics techniques will contribute to identification of the key mutation mechanisms in ALE (Sandberg et al., 2019).

In this review we summarize the applications of ALE in microalgae, analyze the improvement of ALE in microalgal growth, tolerance, metabolism, and substrate utilization, highlighting several typical studies, summarizing some adaptive mechanisms, and discussing the future direction of ALE in microalgae. The coupling strategy of ALE and microalgae may provide a cost-effective, efficient approach for the practical industrial application of microalgae.

## ALE Principle and Experimental Design

In the preceding ALE studies, researchers often used the terms acclimation and adaptation indiscriminately. Ownby et al. (2002) defined these two terms in the study on the tolerance of fish (*Fundulus heteroclitus*) to creosote-contaminated sediments. Recent studies on ALE also try to provide a clear definition (Kleine et al., 2021; LaPanse et al., 2021). In conclusion, acclimation is a type of phenotypic plasticity, not heritable, that organisms adapt to the environment rapidly in a short time; adaptation involves the acquisition or recombination of genetic traits, which can enhance the phenotype of multiple generations to applied stress.

Mutations are the basis for acquiring beneficial mutants through ALE. According to previous studies, experimental measures of mutation rates in microalgae range from 10^–5^ to 10^–10^ mutants per cell per generation (Marvá et al., 2010; Huertas et al., 2011; Perrineau et al., 2014a). Various types of mutations, include transposable element (insertional sequence, IS), single-nucleotide polymorphisms (SNPs), and insertions and deletions (InDels). By integrating a type-II Gulliver-related transposable element into the B_12_-independent methionine synthase gene (METE) during ALE, *C. reinhardtii* became B_12_-dependent in fewer than 500 generations of growth in the presence of vitamin B_12_ (Helliwell et al., 2015). After 1880 generations of ALE, 1937 polymorphic DNA regions were found, among which 149 SNPs resulted in nucleotide substitution in the fast-growing *C. reinhardtii* genome (Perrineau et al., 2014a). Changes in these genomes can alter the metabolic pathways of mutant strains, making it easier for the resulting strains to adapt to the imposed environmental conditions. The difference between ALE and natural selection is that ALE can obtain target phenotypes by controlling specific environmental conditions. In the early stage of ALE, mutants induced by specific conditions began to appear in the cultures. Over time, these beneficial mutants gradually replaced the original algal strain, eventually becoming the dominant strain.

**FIGURE 1:**
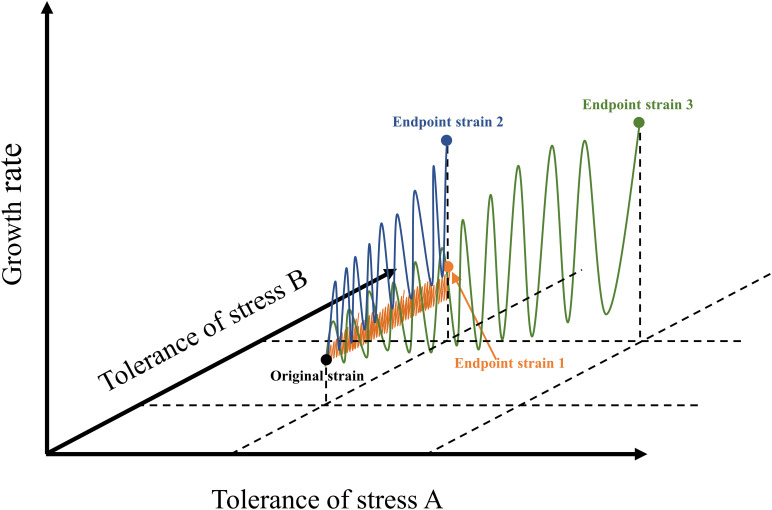
Schematic diagram of different microalgal ALE approaches. The blue line represents ALE pattern of microalgae under appropriate stress concentration; the orange line indicates the ALE pattern of microalgae under low stress concentration; the green line represents the possible ways in which ALE of microalgae could be applied to two stress conditions simultaneously. This figure was modified according to the research (Zhao and Huang, 2020).

At present, the number of ALE studies on freshwater species is much greater than that on marine species (Supplementary Table 1). Several studies on the ALE of microalgae under synthetic wastewater (Rezaei et al., 2019), municipal secondary-treated wastewater (Osundeko et al., 2014), light intensity stress (Deblois et al., 2013), and nitrogen stress (Li T. et al., 2016) proved that the effect of ALE on microalgal fitness is species-specific. Unlike a chemostat, which automatically performs continuous adaptive evolution, as commonly used in bacteria and fungi, microalgae perform ALE frequently by way of a serial transfer procedure. Each transfer procedure is considered to have completed a cycle, and one generation indicates that the number of microalgal cells has doubled. In general, a single cycle of ALE of microalgae lasted from 1 to 30 days. The applied stress conditions limited the density of algal cells at the end of each cycle, resulting in a lower number of generations in each cycle. Undoubtedly, the number of generations is more important than cycle when reaching a more stable adaptive evolutionary algal strains, but this datum is usually lacking in most cases, and should be elucidated in future research (Supplementary Table 1). In fact, the selection of environmental stress determines the efficiency of ALE to a certain extent (Figure 1). If the environmental stress exceeds the tolerance of microalgae, microalgae cannot survive, it will lead to the failure of ALE (Bautista-Chamizo et al., 2018). If the intensity of environmental stress is very low, the efficiency of ALE will be low, and the whole process will be time-consuming (Cheng et al., 2019). Similarly, the design of cycle times of ALE also affects the evolution efficiency and robustness of microalgae. For instance, the ALE of *C. reinhardtii* in TAP medium increased the biomass concentration of the endpoint strains of cc4324, cc4326, and cc4334, which gradually appeared after 15 cycles (Yu et al., 2013). Furthermore, the results of several studies indicated that the biomass concentration of different species of microalgae increased gradually after 15 or 16 cycles during the evolution, indicating that the growth of microalgae was unstable during the early stage of ALE (Fu et al., 2012, 2013; Yu et al., 2013).

To optimize the fitness of microalgae under multiple stresses, some studies have examined two-step ALE: after 210 cycles of ALE with sethoxydim, sesamol-based ALE (over 100 cycles) was undertaken, to increase DHA productivity of *C. cohnii* (Diao et al., 2019). In addition, *Chlorella* sp. AE10 with high CO_2_ tolerance was obtained by implementing ALE (Li et al., 2015). Thereafter, the second ALE was implemented to improve the tolerance of *Chlorella* sp. AE10 in a high-salinity environment (30 g/L NaCl) (Li X. et al., 2018). Besides, a novel two-step ALE method combining ^60^Co-γ irradiation (Cheng et al., 2016) and high concentrations of CO_2_ (Cheng et al., 2016; Kato et al., 2017) has also been proposed to improve the efficiency of ALE. Most studies only apply a single stress condition at any time to improve the tolerance of microalgae to the stress exerted, but practical application conditions are far less suitable for the growth of microalgae than laboratory conditions, and multiple stresses may exist at the same time. Accordingly, multiple stress conditions can be applied simultaneously in an ALE experiment to improve the efficiency of ALE to cope with the complex environmental conditions encountered in practice. Unfortunately, only three studies describe the application of cooperative two-factor ALE strategy: one to optimize the lipid productivity of *Schizochytrium* sp. in combination with low temperature and high salinity (Sun et al., 2018b), another to improve growth and tolerance of *Emiliania huxleyi* under CO_2_ and high-temperature stresses (Schlüter et al., 2014), and the other to promote growth of *Thalassiosira weissflogii* by combining high temperature and high CO_2_ concentration at the expense of inhibiting photochemical performances (Zhong et al., 2021).

Part of the current research is to determine an ALE time endpoint, and some terminate the experiment based on changes in the phenotype of the algal strain. Although ALE can be implemented indefinitely, for practical application, it is necessary to determine a reasonable ALE endpoint by comparing the potential benefits of algal fitness with the time and economic cost of ALE. In addition, modeling methods have been used to optimize the ALE on bacteria and determine the optimal endpoint (Bittihn et al., 2017; LaCroix et al., 2017), which will also be a dilemma that needs to be solved in a subsequent study.

## ALE Application in Enhancing Microalgal Growth

The industrial application of microalgae commonly under the abiotic stress, such as nitrogen stress, high light intensity, and osmotic stress, may increase the content of high value-added metabolites in microalgae. Nonetheless, the stresses typically inhibit the growth of microalgae, by that reducing their overall productivity, which increases the cost of microalgae technology application. ALE is a stress-induced procedure to optimize the tolerance of microalgae to the applied stress. Accordingly, ALE has been applied to optimize the growth of varied industrially related microalgae, to optimize the growth of microalgae under specific conditions, and to reveal the latent mechanism of ALE in microalgal growth optimization (Yu et al., 2013; Wang et al., 2016; Li X. et al., 2017; Kim et al., 2021; Li et al., 2021). Since the growth rate of microalgae is a primary consideration for the successful application of microalgae related biotechnology, the characterization of microalgal growth is usually one of the indicators evaluated in an ALE. For example, Shin et al. (2017) in the process of maintaining the growth of *Chlamydomonas reinhardtii* CC-124 for 5 years, inadvertently conducted an ALE experiment, and isolated a mutant strain with increased growth rate and lipid production. This rapid-growth phenotype was achieved through non-synonymous substitution to 33 genes which will be involved in cell cycle, division, or proliferation. Another study indicated that *C. reinhardtii* CC-503 evolved after 1880 generations under continuous illumination, the growth rate of evolved strain was 1.35 times that of the original strain, which was caused by 149 single nucleotide polymorphisms issuing non-synonymous amino acid substitutions and up-regulation of genes involved in protein synthesis, the cell cycle, and cellular respiration (Perrineau et al., 2014a). The results of these two cases suggest that continuous illumination can induce the strains derived from *C. reinhardtii* to achieve rapid growth rates via non-synonymous substitutions and up-regulation of gene expression related to cell proliferation.

ALE has been used to improve growth capabilities of *Haematococcus pluvialis* under 15% CO_2_ (Cheng et al., 2016). Transcriptome sequencing analysis indicated that ALE enhanced photosynthesis, carbon fixation, and glycolysis pathway of *H. pluvialis*. The up-regulation of *PetH* (ferredoxin–NADP^+^ reductase) in electron transportation, *ATPF0A* (F-type H^+^-transporting ATPase subunit a) in ATP synthase, and *PetJ* (cytochrome c6) in NADPH generation promoted photosynthesis. The up-regulation of genes of C3 and C4 pathways (*PddK*, pyruvate, orthophosphate dikinase; *FBA*, fructose-bisphosphate aldolase, class I) helped increase carbon fixation under a high concentration of CO_2_ (Li K. et al., 2017).

## ALE Application in Improving Stress Resistance of Microalgae

ALE has been proved to improve the tolerance of microalgae to stress. ALE researchers have revealed the adaptive effects under various conditions: flue gas (mainly includes CO_2_, NO*_*x*_*, and SO*_*x*_*) (Cheng et al., 2019), ultraviolet radiation (UVR) (Korkaric et al., 2015), light intensity (Deblois et al., 2013), organic contaminants (Cho et al., 2016; Wang et al., 2016; Li et al., 2021), wastewater (the main stressors were high concentrations of heavy metals, oxygen, and ammonium nitrogen) (Osundeko et al., 2014), landfill leachate (the main stressors were high concentrations of heavy metals, and ammonium nitrogen) (Okurowska et al., 2021), heavy metals (Muyssen and Janssen, 2001; Mikulic and Beardall, 2014; Yu et al., 2020), nutrient stress (Helliwell et al., 2015; Li T. et al., 2016), sludge extract (Wang et al., 2018), CO_2_ (Collins and Bell, 2004; Lohbeck et al., 2012; Schlüter et al., 2014; Li et al., 2015; Cheng et al., 2016; Schaum et al., 2017; Listmann et al., 2020), and high salinity (Perrineau et al., 2014b; Lachapelle et al., 2015; Kato et al., 2017; Meijer et al., 2017; Li X. et al., 2018; Sun et al., 2018a; Hu et al., 2020). The cultivation of microalgae under abiotic stress conditions may increase the synthesis of high-value metabolites. However, these compounds generally inhibit cell growth, thereby reducing the overall productivity of microalgae and increasing the cost of production. As a result, the efficiency of practical application of most microalgae-based engineering techniques is often limited due to abiotic stress. For example, the toxicity of high concentrations of phenol prevents algae from being able to degrade phenol, which limits the application of microalgae-based bioremediation technology in the case of phenol leakage. Li et al. (2021) addressed this with an ALE experiment on *Isochrysis galbana* in 200 mg/L phenol, wherein a strain was obtained that had improved not just the tolerance to phenol but also the removal efficiency thereof. This indicates that pressure amelioration by ALE can modify multiple performance of the evolved strain simultaneously.

Through comparative transcriptomic analysis, genes that are selectively regulated under ALE conditions can be identified, and the mechanism of tolerance improvement can be inferred. A study by Zhou et al. (2017) stands as an explicit example of ALE results explicating the molecular mechanisms of adaptation: a *Chlorella* strain was evolved for enhanced phenol tolerance, the final biomass of the resulting strain was 1.5 times higher than that of the original strain under 500 mg/L phenol (Wang et al., 2016). The improvement of the tolerance of evolutionary strains obtained by ALE under a single stress is consistently specific to the applied stress. Using a complex mixture such as flue gas (Cheng et al., 2019), wastewater (Osundeko et al., 2014; Rezaei et al., 2019), and landfill leachate (Okurowska et al., 2021) as a complex stress condition for ALE can improve the tolerance of microalgae to different components in these mixtures simultaneously. Comparative transcriptomic analysis revealed that the genes related to superoxide dismutase (SOD), ascorbate peroxidase (APX), catalase (CAT), glutathione reductase (GR), and carotenoid biosynthesis were significantly up-regulated by 500 mg/L phenol ALE compared with the original *Chlorella* sp. L5 strain, indicating that ALE can allow *Chlorella* sp. L5 to exhibit stronger defense against antioxidants (Zhou et al., 2017).

It is worth noting that not all ALE experiments can improve the tolerance of microalgae, as has been shown in ALE of *Nitzschia closterium* and *Chlorella* sp. 12 using Cu (Johnson et al., 2007). This may be because the selected Cu concentration is too low, resulting in the low evolutionary efficiency, which is not enough to improve the tolerance to any significant extent within the duration of the experiment. Inversely, ALE with single stress can be used to enhance tolerance to multiple stress simultaneously, as proved by a study in which *C. reinhardtii* CC125 strain was evolved for enhanced tolerance to both UVR and rose Bengal (Korkaric et al., 2015).

## ALE Application in Regulating Microalgal Metabolic Pathways for Producing Biologically Active Substances

Maximizing the yield of valuable target metabolites is an important issue needing to be addressed in microalgal biotechnology applications (Sun et al., 2016; Zhao et al., 2019; Deka et al., 2020). Reasonable ALE cannot only improve the tolerance of microalgae to abiotic stress, but also optimize the yield of target metabolites. ALE can increase the synthesis of high-value metabolites, including docosahexaenoic acid (DHA) (Li X. et al., 2017; Sun et al., 2018b; Diao et al., 2019; Gachelin et al., 2020; Hu et al., 2021), eicosapentaenoic acid (EPA) (Wang et al., 2019), carbohydrates (Cheng et al., 2019), carotenoid (Fu et al., 2013; Yi et al., 2015; Cheng et al., 2016; Han et al., 2019). Furthermore, the production of biofuel and high value-added by-products from ALE will improve the economic feasibility of biofuel production and microalgal biorefineries. ALE used to phenol up-regulated genes related to fatty acid and starch biosynthesis pathways, resulting in higher total carbohydrate and lipid contents than the original strain (Zhou et al., 2017). Use of glucose for ALE could increase the accumulation of DHA-rich lipids, up-regulate the hub metabolites of glycerol, glutamic acid, malonic acid, and succinic acid, and improve the negative regulation of tyrosine, fructose and lyxose (Li X. et al., 2017). By implementing ALE, *Schizochytrium* sp. HX-308 was evolved to increase the metabolic flux of fatty acid synthase pathway and inhibit the metabolism of polyketide synthase pathway, resulting in a 53% higher lipid content than that when using the original strain (Sandberg et al., 2019). After ALE with 15% CO_2_, the up-regulation of *FBA*, *TPI* (triosephosphate isomerase), and *PK* (pyruvate kinase) increased the glycolysis pathways. This up-regulation aids conversion of photosynthetic carbon to pyruvate (an imperative precursor for astaxanthin and lipid synthesis) (Li K. et al., 2017).

Chemical modulators can perturb the biological system and induce cells to metabolize desired chemicals by specifically targeting enzyme proteins or as signaling molecules (Carlson and White, 2012). This strategy can be used to improve microalgal lipid production, but the direct addition of chemical modulators to the medium has obvious negative effects on biomass accumulation or lipid synthesis (Wase et al., 2017). Even so, chemical modulator-based ALE has also been profitably operated, as in a study by Diao et al. (2019) wherein the addition of acetyl-CoA carboxylase (ACCase) inhibitor could redirect carbon equivalents from starch to lipid. After sesamol-based ALE, the lipid and DHA productivities of *Crypthecodinium cohnii* were doubled (Diao et al., 2019). Such approaches can be used to optimize the metabolites of microalgae in various industrial applications.

Although the ability of ALE to enhance the desired microalgal metabolite production has been demonstrated, the study still indicated that there was no difference in lipid productivities between a *Chlorococcum littorale* strain grown after eight cycles of short-term nitrogen starvation and a control group (Cabanelas et al., 2016). The results indicated that suitable algal strains and an appropriate ALE method were also important factors to improve the metabolic yield. As the understanding of metabolic and regulatory networks continues to increase, ALE will be increasingly used as a complementary technique for optimizing high-value-added microalgae.

## ALE Application in Promoting Biodegradation of Pollutants by Microalgae

For some industrial applications such as wastewater treatment, flue gas treatment, and bio-enhanced degradation of hazardous chemicals it is necessary for microalgae to make effective use of substrates. The primary motivation of improving the substrate utilization by microalgae is usually related to the treatment of pollutants (Acuner and Dilek, 2004). The removal rate of 10 mg/L levofloxacin (LEV) by *Chlorella vulgaris* was increased by 67%, and the half-life was shortened by 92 days, after ALE with 200 mg/L levofloxacin (Xiong et al., 2017). Phenol is the most commonly used organic contaminant for ALE of microalgae. Using 100–500 mg/L phenol for ALE can improve the removal rate of phenol and shorten its half-life in the environment when using *Chlorella* sp. (Wang et al., 2016) and *I. galbana* Parke (Li et al., 2021). ALE has been used in a trouble-shooting role in the utilization of municipal secondary-treated wastewater (Osundeko et al., 2014), sludge extracts (Wang et al., 2018), and synthetic wastewater (Rezaei et al., 2019) as a nutrient source, and can improve the removal ability of microalgae by through metabolic selection. In conclusion, ALE is suitable for use in microalgae-based wastewater treatment and cultivation without, or with reduced, dilution. Particularly, ALE successfully enhanced the removal rates of SO*_*x*_*, and NO*_*x*_* by up-regulating genes related to extracellular sulfur transport and nitrate reductase in *Chlorella* sp. (Cheng et al., 2019).

## Summary

ALE is a promising approach to improve the phenotype of microalgae based on random mutation and natural selection. Moreover, ALE can improve the tolerance of microalgae to the selected stress conditions and the rate of substrate utilization and provide technical support for the actual production of microalgal metabolites and microalgae-based enhanced bioremediation of pollutants. Evolved strains induced by ALE have been demonstrated to activate latent metabolic pathways, enhance substrate tolerance, and improve the fitness of microalgae.

ALE also has some limitations: it can only achieve phenotype optimization through mutation and evolution; the evolved microalgal strains may have some side effects while improving the fitness of selection (e.g., overproduction of unnecessary metabolites, and elimination of the original fitness). Besides, the stability and reversibility of ALE resulting strains are generally not mentioned. Therefore, it is also a crucial part of the future study to verify the long-term stability of ALE resultant strains. For the design of ALE experiment, the evolution time, cycle frequency, and stress selection are the key factors for the success of ALE. In industrial/practical application, the appropriate ALE time should be selected. If ALE is too long, the modification of phenotype will gradually become insignificant, rendering the ALE uneconomic.

In conclusion, improving the efficiency of ALE is the primary problem to be solved in future research. It may be useful to solve this problem by applying multiple, simultaneous stress conditions on ALE in microalgae. As a result, ALE improves the tolerance of algal strains, optimizes the metabolites, and increases the rate of utilization of pollutants, making it a promising method for the application of microalgae biotechnology.

## Author Contributions

BZ drafted the manuscript. All authors contributed significantly to improve the manuscript through intensive discussion and substantial additions and revisions of passages in the text. All authors contributed to the article and approved the submitted version.

## Conflict of Interest

The authors declare that the research was conducted in the absence of any commercial or financial relationships that could be construed as a potential conflict of interest.

## Publisher’s Note

All claims expressed in this article are solely those of the authors and do not necessarily represent those of their affiliated organizations, or those of the publisher, the editors and the reviewers. Any product that may be evaluated in this article, or claim that may be made by its manufacturer, is not guaranteed or endorsed by the publisher.
