# Supplementary material for: Adaptive Laboratory Evolution of Microalgae: A Review of the Regulation of Growth, Stress Resistance, Metabolic Processes, and Biodegradation of Pollutants
Source: Front Microbiol. 2021 Aug 18;12:737248. doi: 10.3389/fmicb.2021.737248 (PMC8416440; doi:10.3389/fmicb.2021.737248)
Supplement: Supplementary file 1 [file Table_1.DOCX]

Supplementary Material

**Table S1.** Application of ALE to microalgae via various stress conditions.

| Microalgal species | ALE conditions | ALE intensities | Time for ALE (d) | Number of cycles | Cycle period (d) | Generations | Growth | Stress resistance | Metabolites | Mechanism | References |  |
| --- | --- | --- | --- | --- | --- | --- | --- | --- | --- | --- | --- | --- |
|  | ***Organic contaminants*** | |  |  |  |  |  |  |  |  |  |  |
| *Dunaliella salina* CCAP 19/20 (MS) | Phenol | 0, 50, 100 or 150 mg/L | 10 | 1 | 10 | / | + | Nd | Lipid productivity +, C18:3(n-3), C18:3(n-6) +, C17:1 -, C18:0 - | Nd | (Cho et al., 2016) |  |
| *Chlorella* sp. | Phenol | 500 mg/L | ~95 | 31 | ~3 | / | + | + | Total carbohydrate +, Total protein -, Total lipid + | Comparative transcriptomic analysis: + antioxidant enzymes (SOD, APX, CAT and GR) and carotenoids (astaxanthin, lutein and lycopene); + PS I, PS II, photosynthetic electron transport chain and starch biosynthesis | (Wang et al., 2016; Zhou et al., 2017) |  |
| *Isochrysis galbana* Parke MACC/H59 (MS) | Phenol | 100 or 200 mg/L | 90 | 30 | 3 | / | + | + | Phenol hydroxylase + | Nd | (Li et al., 2021) |  |
|  | ***Herbicides*** | |  |  |  |  |  |  |  |  |  |  |
| *Dictyosphaerium chlorelloides* | Simazine | 3.1 μg/L | 60 | 12 | 5 | / | - | + | Nd | Spontaneous mutations | (Marvá et al., 2010) |  |
| *Scenedesmus intermedius* | Simazine | 3.1 μg/L | 60 | 12 | 5 | / | - | + | Nd | Spontaneous mutations | (Marvá et al., 2010) |  |
| *Scenedesmus intermedius* | Diquat | 120 μg/L | 60 | 12 | 5 | / | - | + | Nd | Pre-selective mutations | (Marvá et al., 2010) |  |
|  | ***Dye*** | |  |  |  |  |  |  |  |  |  |  |
| *Chlorella vulgaris* | Tectilon yellow 2G (TY2G) | 50, 200, 400 mg/L | 19 | 1 | 19 | / | Nd | Nd | Nd | Nd | (Acuner and Dilek, 2004) |  |
|  | ***Antibiotic*** | |  |  |  |  |  |  |  |  |  |  |
| *Chlorella vulgaris* | Levofloxacin | 200 mg/L | 11 | 1 | 11 | / | = | = | Nd | Nd | (Xiong et al., 2017) |  |
|  | ***Trace metal*** | |  |  |  |  |  |  |  |  |  |  |
| *Nitzschia closterium* (MS) | Cu | 5 or 25 μg/L | 200 | / | / | / | = | = | Nd | Nd | (Johnson et al., 2007) |  |
| *Chlorella* sp. 12 | Cu | 2 μg/L | 100 | / | / | / | = | = | Nd | Nd | (Johnson et al., 2007) |  |
| *Chlamydomonas reinhardtii* CC125 | Cd | 0.05 and 0.5 μM | 420 | 140 | 3 | / | + | + | Nd | Whole-genome re-sequencing and transcriptome analysis: mutations of genes involved in photosynthesis (*PSBP1*), glutathione metabolism (*CHLREDRAFT_167073*, *GPX5*) and calcium transport | (Yu et al., 2020) |  |
| *Raphidocelis subcapitata* | Zn | 65 μg/L | 100 | / | / | / | + | + | Nd | Nd | (Muyssen and Janssen, 2001) |  |
| *Chlorella vulgaris* | Zn | 65 μg/L | 110 | / | / | / | + | + | Nd | Nd | (Muyssen and Janssen, 2001) |  |
| *Chlamydomonas reinhardtii* | Zn | 4.6 × 10^-4^ mg/L | / | / | / | >80 | Nd | = | Nd | Nd | (Mikulic and Beardall, 2014) |  |
| *Cyanidium caldarium* | Zn | 3.9 mg/L | / | / | / | >80 | Nd | + | Nd | Nd | (Mikulic and Beardall, 2014) |  |
|  | ***Waste water*** | |  |  |  |  |  |  |  |  |  |  |
| *Chlamydomonas debaryana*;  *Chlorella luteoviridis*;  *Chlorella vulgaris*;  *Desmodesmus intermedius*;  *Hindakia tetrachotoma*;  *Parachlorella kessleri*; | Municipal secondary-treated wastewater (MSTW) | 100% | 56 | 8 | 7 | / | + | + | Total chlorophyll content +, Ascorbate peroxidase activity +, Carotenoid + | Nd | (Osundeko et al., 2014) |  |
| *Arthrospira platensis* | Synthetic secondary wastewater | 100% | 4 | 1 | 4 | / | + | + | Chl-a - | Nd | (Rezaei et al., 2019) |  |
| *Chlorella vulgaris* | Synthetic secondary wastewater | 100% | 4 | 1 | 4 | / | + | + | Chl-a - | Nd | (Rezaei et al., 2019) |  |
| *Chlorella vulgaris* | Sludge extracts | 25%, 50%, 75% or 100% | 24 | 3 | 8 | / | + | + | Nd | Proteomics analysis: + pyruvate fermentation, TCA cycle, and glycolysis after acclimation | (Wang et al., 2018) |  |
| *Chlorella vulgaris* | Domestic landfill leachate | 20% (v/v) | ~730 | ~35 | ~21 | / | + | + | Nd | Nd | (Okurowska et al., 2021) |  |
|  | ***Flue gas, CO_2_ and acidification*** | |  |  |  |  |  |  |  |  |  |  |
| *Chlorella* sp. AE10 | Flue gas | 10% CO_2_, 200 ppm NOx and 100 ppm SOx; | 138 | 46 | 3 | 110 | + | + | Nd | Comparative transcriptomic analysis: + photosynthesis, oxidative phosphorylation, CO_2_ fixation, sulfur metabolism and nitrogen metabolism | (Cheng et al., 2019) |  |
|  |  |  |  |  |  |  |  |  |  |  |  |  |
|  |  |  |  |  |  |  |  |  |  |  |  |  |
|  |  |  |  |  |  |  |  |  |  |  |  |  |
|  |  |  |  |  |  |  |  |  |  |  |  |  |
| *Haematococcus pluvialis* (mutant) | CO_2_ | From 2% to 15% | 16 | 4 | 4 | >10 | + | + | Astaxanthin yield + | Transcriptome analysis: + photosynthesis, carbon fixation, glycolysis pathways related genes | (Cheng et al., 2016; Li et al., 2017a) |  |
|  |  |  |  |  |  |  |  |  |  |  |  |  |
|  |  |  |  |  |  |  |  |  |  |  |  |  |
|  |  |  |  |  |  |  |  |  |  |  |  |  |
| *Emiliania huxleyi* (MS) | CO_2_ | 1250 ppm | 288 | 36 | 8 | ~200 | = | + | Nd | Nd | (Listmann et al., 2020) |  |
| *Chaetoceros affinis (MS)* | CO_2_ | 1250 ppm | 288 | 36 | 8 | ~200 | = | + | Nd | Nd | (Listmann et al., 2020) |  |
| *Chlorella* sp. | CO_2_ | 10 or 20%CO_2_ | 97 | 31 | ~3 | / | + | + | Carotenoids +, carbonhydrate =, protein =, lipid = | Nd | (Li et al., 2015) |  |
| *Emiliania huxleyi* (MS) | CO_2_ | 400, 1100 or 2200 μatm | 320 | 64 | 5 | ~500 | + | + | Nd | qRT-PCR: + pH regulation, carbon transport genes | (Lohbeck et al., 2012; 2014) |  |
| *Chlamydomonas reinhardtii* | CO_2_ | 430-1050 ppm | ~438 | 125 | 3/4 | ~1000 | = | = | Chlorophyll content + | Nd | (Collins and Bell, 2004) |  |
|  |  |  |  |  |  |  |  |  |  |  |  |  |
| *Thalassiosira weissflogii* (MS) | CO_2_ | 400 or1000 μatm | 224 | 32 | 7 | 330-372 | = | = | Fv/Fm =; rETRmax =; α =; NPQ_max_ + | Nd | (Zhong et al., 2021) |  |
| *Chlamydomonas reinhardtii* | CO_2_ | 400 or1000 ppm | 720 | 180 | 4 | 1326 | + | + | Fv/Fm + | Short-term: metabolic analysis: + FA and amino acids Long-term: metabolic analysis - photosynthesis, glycolysis, lipid biosynthesis, and nitrogen assimilation; + TCA cycle and β-oxidation | (Zhang et al., 2021) |  |
| *Tetraselmis chuii,* (MS) | pH (acidification scenarios) | 7.4 or 6.0 | 16 | 4 | 4 | 4 | = | = | Chl-a - | Nd | (Bautista-Chamizo et al., 2018) |  |
| *Phaeodactylum tricornutum* (MS) | pH (acidification scenarios) | 7.4 or 6.0 | 16 | 4 | 4 | 4 | - | - | Chl-a - | Nd | (Bautista-Chamizo et al., 2018) |  |
|  | ***Salinity*** | |  |  |  |  |  |  |  |  |  |  |
| *Schizochytrium* sp. HX-308 (MS) | NaCl | 30 g/L | 150 | 150 | 1 | / | + | + | Total lipids +, lipid productivity +, SFA +, PUFA -, ROS -, T-AOC +, MDA - | Comparative transcriptomic analysis: + antioxidant enzymes (SOD1, SOD2, CAT), + central carbon metabolism (ATP citrate lyase, pyruvate dehydrogenase and phosphoenolpyruvate carboxylase), + fatty acid synthase, - polyketide synthase (+ ORFA, - ORFB, - PRFC), + GPAT, + DGAT | (Sun et al., 2018a) |  |
| *Synechocystis* sp. PCC 6803 | NaCl | 3% | 303 | ~43 | 7 | / | + | + | Glucosyl glycerol +, sucrose + | Transcriptomic analysis: + potassium ion transmembrance transport and cellular potassium ion transport related genes | (Hu et al., 2020) |  |
| *Chlorella* sp. AE10 | NaCl | 26-30 g/L | 138 | 46 | 3 | / | - | + | Carbohydrates +, Chl-a -, Chl-b -, carotenoid - | Comparative transcriptomic analysis: + antioxidant enzymes, CO_2_ fixation, amino acid biosynthesis, central carbon metabolism and ABC transporters related genes | (Li et al., 2018) |  |
|  |  |  |  |  |  |  |  |  |  |  |  |  |
| *Chlamydomonas reinhardtii* | NaCl | 24-36 g/L | / | / | 7 | ~500 | + | + | Nd | Nd | (Lachapelle et al., 2015) |  |
| *Chlamydomonas reinhardtii* (CC‐503 cw92 mt+) | NaCl | 200 mM | ~510 | 189 | ~3 | 1255 | + | + | Nd | Transcriptome: - stress response and transcription/translation related genes | (Perrineau et al., 2014b) |  |
| *Chlamydomonas moewusii* | NaCl | 100 mM | 7 | 1 | 7 | / | + | + | Phosphatidylinositol phosphate -, phosphatidylinositol 4,5-bisphosphate - | Nd | (Meijer et al., 2017) |  |
| *Chlamydomonas* sp. JSC4 | Sea salt | 5% to 7% | 252 | 36 | 7 | / | + | + | Starch content +, lipid content - | Transcriptional analysis: inactivation of starch-to-lipid biosynthesis switching | (Kato et al., 2017) |  |
|  |  |  |  |  |  |  |  |  |  |  |  |  |
| *Phaeodactylum tricornutum* (CCMP-2561) (MS) | Reducing salinity | 70% | ~252 | 35 | ~7 | / | Nd | Nd | Total fatty acid +, arachidonic acid +, DHA +, EPA + | Nd | (Wang et al., 2019) |  |
| *Dunaliella salina* (MS) | NaCl | 60 g/L | 84 | 12 | 7 | / | - | + | Total protein =; MDA =; GSH -; T-AOC = | qRT-PCR: = ribosomal genes; + stress defense genes; + photosynthetic pathway genes; + LHCB proteins genes | (Zhu et al., 2020) |  |
| *Parachlorella* sp. KSN1 | NaCl | 10 or 20 g/L | 32 | 8 | 4 | / | + | + | FA content +; SFA-; UFA + | Nd | (Kim et al., 2021) |  |
|  | ***Light intensity*** | |  |  |  |  |  |  |  |  |  |  |
| *Ankistrodesmus falcatus*;  *Pandorina morum*;  *Pediastrum boryanum*;  *Chlamydomonas snowii*;  *Fragilaria crotonensis*;  *Aulacoseira granulata* var. angustissima;  *Phormidium muscicola*;  *Microcystis flosaquae*;  *Aphanizomenon flosaquae*;.*Anabaena spiroïdes*; | Low and high light intensity (LL and HL) | 76 μmol photons m^-2^ s^-1^ (Low); 583 μmol photons m^-2^ s^-1^ (High) | Many weeks | / | / | / | Nd | + | Nd | Nd | (Deblois et al., 2013) |  |
|  |  |  |  |  |  |  |  |  |  |  |  |  |
|  |  |  |  |  |  |  |  |  |  |  |  |  |
|  |  |  |  |  |  |  |  |  |  |  |  |  |
|  |  |  |  |  |  |  |  |  |  |  |  |  |
|  |  |  |  |  |  |  |  |  |  |  |  |  |
|  |  |  |  |  |  |  |  |  |  |  |  |  |
|  |  |  |  |  |  |  |  |  |  |  |  |  |
|  |  |  |  |  |  |  |  |  |  |  |  |  |
| *Chlamydomonas reinhardtii* CC125 | Ultraviolet radiation (UVR) | UVR: 124 ± 5 μmol m^-2^ s^-1^ (18 W/m^2^ (UV-A) and 0.8 W/m2 (UV-B)) | 4 | 1 | 4 | / | Nd | + | Nd | Nd | (Korkaric et al., 2015) |  |
| *Dunaliella salina* (MS) | Blue light | / | 25 | 5 | 5 | / | + | + | Beta-carotene + | Nd | (Han et al., 2019) |  |
| *Phaeodactylum tricornutum* (CCAP 1055/1) (MS) | Combined blue and red LED light | 75% red LED light and 25% blue LED light | 55 | 11 | 5 | / | + | = | Neutral lipid =, Chl-a =, β-carotene =, fucoxanthin + | Nd | (Yi et al., 2015) |  |
| *Dunaliella salina* (MS) | Combined blue and red LED light | 75% red LED light and 25% blue LED light | 80 | 16 | 5 | / | + | Nd | Chl-b +, lutein +, β-carotene + | Nd | (Fu et al., 2013) |  |
| *Chlorella vulgaris* | Red light | 660 nm | 114 | 38 | 3 | / | + | Nd | Total chlorophyll content = | Nd | (Fu et al., 2012) |  |
|  | ***Temperature*** | |  |  |  |  |  |  |  |  |  |  |
| *Galdieria sulphuraria* RT22 | Cold stress | 28°C (lower than optimal growth temperature of 42°C) | 240 | / | / | 102 | + | + | Nd | Nd | (Rossoni and Weber, 2019) |  |
| *Chlamydomonas reinhardtii* | Warm environment | 4 °C above ambient temperature | ~3650 | / | / | / | Nd | + | Nd | Photosynthetic capacity +, photoinhibition susceptibility - | (Schaum et al., 2017) |  |
| *Dunaliella salina*(MS) | Cold stress | 7°C | 5-7 | 1 | 5-7 | / | Nd | + | Nd | Nd | (Zchut et al., 2003) |  |
| *Tisochrysis lutea* (CCMP 463 and RCC 1344) | Oscillating thermal stress | / | 150 | 13 | 7-24 | / | = | Nd | Total lipid +, neutral lipids +, phospholipids +, glycolipids +, DHA +, SFA -, MUFA + | Genomic analysis: mutations corresponding to a total change of genotype | (Gachelin et al., 2020) |  |
|  |  |  |  |  |  |  |  |  |  |  |  |  |
|  |  |  |  |  |  |  |  |  |  |  |  |  |
|  |  |  |  |  |  |  |  |  |  |  |  |  |
|  |  |  |  |  |  |  |  |  |  |  |  |  |
|  |  |  |  |  |  |  |  |  |  |  |  |  |
|  |  |  |  |  |  |  |  |  |  |  |  |  |
| *Thalassiosira weissflogii* (MS) | Warm environment | 25°C | 224 | 32 | 7 | 330-372 | + | + | Fv/Fm =; rETRmax +; α =;NPQ_max_ + | Nd | (Zhong et al., 2021) |  |
| *Schizochytrium* sp. | Warm environment | 34.5°C | 80 | 80 | 1 | / | + | + | DPA +; PUFA +; DHA +; EPA =; Lipid yield +; β-carotene +; astaxanthin -; heat shock protein 20 -; | Nd | (Hu et al., 2021) |  |
|  | Nutrients | |  |  |  |  |  |  |  |  |  |  |
| *Chlorella* strains (*Chlorella* sp. C2, *Chlorella sorokiniana* C3, *Chlorella sorokiniana* C7, *Chlorella pyrenoidosa* (FACHB1216)) | NaNO_2_ | 12.2 g/L | 10 | 1 | 10 | / | Nd | Nd | Triacylglycerols + | Nd | (Li et al., 2016) |  |
| *Micractinium* sp. GA001 (MS) | NaNO_3_ (Nitrogen-depletion condition) | 0.004 g/L | 72 | 3 | 24 | / | + | Nd | Total lipid +, ω3, ω6, C20:0, C20:1, and C20:5 + | Nd | (Deka et al., 2020) |  |
| *Chlorococcum littorale* (NBRC 102761) (MS) | N-starvation | Algal cells can deplete nitrogen in 2 days | 134 | 13 | 6 or 12 | / | = | = | SFA =, MUFA =, PUFA = | Nd | (Cabanelas et al., 2016) |  |
| *Crypthecodinium cohnii* ATCC 30556 (MS) | Glucose | 9-54 g/L | 650 | 260 | 2.5 | / | + | + | Total lipid +, DHA + | Metabolomic analysis: + glycerol, glutamic acid, malonic acid and succinic acid, - tyrosine, fructose and lyxose | (Li et al., 2017b) |  |
| *Chlamydomonas reinhardtii* | Vitamin B_12_ | 1000 ng/L | ~140 | 70 | ~2 | <700 | + | + | Nd | Southern blotting: type-II Gulliver-related transposable element integrated into the B_12_-independent methionine synthase gene (METE) | (Helliwell et al., 2015) |  |
| *Chlamydomonas reinhardtii* (cc4324, cc4326 and cc4334) | TAP | / | 84 | 28 | 3 | / | + | Nd | Lipid content + | Nd | (Yu et al., 2013) |  |
| *Chlamydomonas reinhardtii* CC-124 | TAP | / | ~1825 | ~60 | 30 | / | + | Nd | Total carbohydrate +, FAME yeild + | Genome: 44 coding sequence region alterations, 34 resulted in non‑synonymous substitutions within 33 genes which may mostly be involved in cell cycle, division or proliferation | (Shin et al., 2017) |  |
| *Chlamydomonas reinhardtii* CC-503 | TAP | / | ~510 | 283 | ~2 | 1880 | + | Nd | Nd | Genome: 149 single nucleotide polymorphisms resulting in amino acid substitutions, transcriptome analysis: + protein synthesis, the cell cycle and cellular respiration, - DNA repair pathway and photosynthesis | (Perrineau et al., 2014a) |  |
|  | ***Others*** | |  |  |  |  |  |  |  |  |  |  |
| *Aurantiochytrium* sp. FJU-512 | Sugarcane bagasse hydrolysate (SBH) | 50% to 75% to 100% | 6 | 3 | 2 | 10 | Nd | Nd | Lipid +, DHA + | Transcriptome analysis: + TCA cycle, amino acid biosynthesis, fatty acid metabolism and degradation of aromatic compounds | (Qi et al., 2017) |  |
|  |  |  |  |  |  |  |  |  |  |  |  |  |
|  |  |  |  |  |  |  |  |  |  |  |  |  |
| *Schizochytrium* sp. HX-308 (MS) | High agitation rate (high oxygen) | 230 rpm | 40 | 40 | 1 | / | + | Nd | Lipid -, squalene - | Nd | (Sun et al., 2016) |  |
| *Crypthecodinium cohnii* ATCC 30556 (MS) | Sethoxydim (targeting ACCase) | 10 μM to 60 μM | 525 | 210 | ~2.5 | / | = | Nd | Total lipids +, total fatty acids +, starch -, α-amylase activity +, malonyl-CoA + | qRT-PCR: + UTP-glucose-1-phosphate uridylyltransferase and starch synthase coding genes, + α-glucan water dikinase and α-amylase coding genes; Transcriptional analysis: + lipid biosynthetic pathway | (Diao et al., 2019) |  |
|  |  |  |  |  |  |  |  |  |  |  |  |  |
| *Crypthecodinium cohnii* ATCC 30556 (MS) | Sesamol | 0.5 mM to 2 mM | 300 | 100 | 3 | / | Nd | Nd | Total lipids + | Quantitative metabolomics: + glycolysis and pentose phosphate pathway | (Diao et al., 2019) |  |
|  | ***Multiple stress conditions*** | |  |  |  |  |  |  |  |  |  |  |
| *Schizochytrium* sp. HX-308 (MS) | Low-temperature + high-salinity | 4°C + 30 g/L NaCl | 90 | 30 | 3 | / | + | + | DHA +, EPA +, DPA +, PUFA +, SFA -, lipid +, T-AOC +, MDA - | qRT-PCR: + SOD, CAT, ORFA, ORFB, and ORFC, - APX and FAS | (Sun et al., 2018b) |  |
| *Emiliania huxleyi* (MS) | CO_2_+high temperature | 400, 1100 or 2200 μatm; 26.3°C | 365 | 73 | 5 | ~460 | + | + | Nd | Nd | (Schlüter et al., 2014) |  |
| *Thalassiosira weissflogii* (MS) | Warm environment +CO_2_ | 25°C +1000 μatm | 224 | 32 | 7 | 330-372 | + | + | Fv/Fm -; rETRmax -; α -; NPQ_max_ - | Nd | (Zhong et al., 2021) |  |

Nd, not detected; /, not mentioned; +, increased; = sustained; −, decreased; FA, fatty acids; SFA, saturated fatty acids; UFA, unsaturated fatty acids; PUFA, polyunsaturated fatty acids; ROS, reactive oxygen species; T-AOC, total antioxidant capacity; MDA, malondialdehyde; GSH, glutathione; EPA, eicosapentaenoic acid; DHA, docosahexaenoic acid; DPA, docosapentaenoic acid; FAME, fatty acid methyl ester; Fv/Fm, maximal photochemical quantum yield of PSII; rETRmax, maximal relative electron transport rate; α, apparent photosynthetic efficiency; NPQmax, maximal non-photochemical quenching;
